# Context awareness based Sketch-DeepNet architecture for hand-drawn sketches classification and recognition in AIoT

**DOI:** 10.7717/peerj-cs.1186

**Published:** 2023-04-27

**Authors:** Safdar Ali, Nouraiz Aslam, DoHyeun Kim, Asad Abbas, Sania Tufail, Beenish Azhar

**Affiliations:** 1 Department of Software Engineering, University of Lahore, Lahore, Punjab, Pakistan; 2 Department of Computer Engineering, Jeju National University, Jeju, Jeju, South Korea; 3 Department of Computer Science, University of Central Punjab, Lahore, Punjab, Pakistan

**Keywords:** Convolutional neural networks (CNNs), Deep neural networks (DNNs), Sketch recognition, TU-Berlin

## Abstract

A sketch is a black-and-white, 2-D graphical representation of an object and contains fewer visual details as compared to a colored image. Despite fewer details, humans can recognize a sketch and its context very efficiently and consistently across languages, cultures, and age groups, but it is a difficult task for computers to recognize such low-detail sketches and get context out of them. With the tremendous increase in popularity of IoT devices such as smartphones and smart cameras, *etc*., it has become more critical to recognize free hand-drawn sketches in computer vision and human-computer interaction in order to build a successful artificial intelligence of things (AIoT) system that can first recognize the sketches and then understand the context of multiple drawings. Earlier models which addressed this problem are scale-invariant feature transform (SIFT) and bag-of-words (BoW). Both SIFT and BoW used hand-crafted features and scale-invariant algorithms to address this issue. But these models are complex and time-consuming due to the manual process of features setup. The deep neural networks (DNNs) performed well with object recognition on many large-scale datasets such as ImageNet and CIFAR-10. However, the DDN approach cannot be carried out for hand-drawn sketches problems. The reason is that the data source is images, and all sketches in the images are, for example, ‘birds’ instead of their specific category (*e.g*., ‘sparrow’). Some deep learning approaches for sketch recognition problems exist in the literature, but the results are not promising because there is still room for improvement. This article proposed a convolutional neural network (CNN) architecture called Sketch-DeepNet for the sketch recognition task. The proposed Sketch-DeepNet architecture used the TU-Berlin dataset for classification. The experimental results show that the proposed method beats the performance of the state-of-the-art sketch classification methods. The proposed model achieved 95.05% accuracy as compared to existing models DeformNet (62.6%), Sketch-DNN (72.2%), Sketch-a-Net (77.95%), SketchNet (80.42%), Thinning-DNN (74.3%), CNN-PCA-SVM (72.5%), Hybrid-CNN (84.42%), and human recognition accuracy of 73% on the TU-Berlin dataset.

## Introduction

Nowadays, we find sketches in many aspects of daily life, and they play a significant role in human-computer interaction, education, recording, and suspect identification. AIoT-based systems can be built in order to obtain maximum benefits from hand-drawn sketches which allow the user to first draw the sketch on any touch-screen device, and then the drawn sketch can be used to search for the object over the internet or understand the context in the case of multiple sketches in AIoT based systems. The first challenge in implementing such systems is to develop an accurate and useful sketch classification model.

The main purpose of sketch classification is to properly identify the class or label of the drawn object over a pre-determined set of classes. For this, it is important to find distinctive and powerful features of the given sketch image. Previous work on sketch classification generally utilized the image classification approach by extracting hand-crafted features from sketch images and then feeding them to a classifier. Existing approaches which is based on the handcrafted feature extraction methodology are HOG (Dalal & Triggs, 2005), SIFT (Lowe, 2004), SSIM (Shechtman & Irani, 2007), fisher vector (Schneider & Tuytelaars, 2014), and GF-HOG (Hu et al., 2013). These feature descriptors are often combined with a bag of visual words (BoW) (McCallum & Nigam, 1998; Joachims, 1998) to yield final features for the classification purpose. However, these features are sensitive concerning the user point of view. Additionally, the ability of algorithms to train the classification models is also affected by the hand-crafted features. Also, it is nearly impossible to manually identify the number of edges and corners representing good features for a dataset that consists of 250 categories of sketches. Therefore, there is still significant room to improve the classification accuracy of hand-drawn sketches because the accuracy of the existing techniques is not attractive.

Recently, CNN, a specific type of DNN architecture, has been proposed in many computer vision, artificial intelligence, and machine learning fields. CNN architectures, which in essence is a multi-layered neural network model, appear as a powerful framework for features representation and classification in many image processing domains. The CNN features used for classification give a great performance on image datasets (Krizhevsky & Hinton, 2009; Krizhevsky, Sutskever & Hinton, 2012). These features are from CNN layers. CNN architectures consist of multiple layers depending on the domain targeted. These well-known layers are the convolutional, pooling, normalization, and fully connected layers. In CNN architectures, various layers provide different representations of input data. For the classification of large image datasets such as CIFAR-10/100 (Krizhevsky & Hinton, 2009), ImageNet (Deng et al., 2009), and MNIST (LeCun et al., 1998), CNN has been used. The domain of sketches classification *via* CNN required more attention. With the arrival of large-scale datasets such as Sketchy (Sangkloy et al., 2016) and TUBerlin (Eitz, Hays & Alexa, 2012), CNN received more attention in sketch classification (Dai et al., 2017; Yang & Hospedales, 2015; Yu et al., 2015, 2017; Sarvadevabhatla & Babu, 2015).

It is a challenging task for computer systems to recognize hand-drawn sketches automatically or without human intervention. There are two fundamental reasons behind this. First, sketches are abstract and contain little details about the object’s shape and appearance, and second, variation in sketching style, *e.g*., everyone draws the same object differently. So, it is difficult for state-of-the-art algorithms to classify the hand-drawn sketches effectively and with acceptable accuracy.

This article presents a deep convolutional neural network (DCNN) based sketch classification architecture named Sketch-DeepNet across numerous object categories such as cat, car, apple, *etc*. The proposed architecture consists of seven layers. CNN chooses features in two ways, either manually or automatically. This article made it automatically by designing an automatic CNN-based network to extract discriminative features from training data to perform classification. Particularly, the proposed method included random neural node drop-out and utilized fewer convolutional layers. The fewer use of convolutional layers decreases the number of parameters in the CNN model to avoid the over-fitting problem. The proposed CNN model achieves 95.05% accuracy on the TU-Berlin sketch dataset which is much better as compared to DeformNet (62.6%) (Dai et al., 2017), Sketch-DNN (72.2%) (Yang & Hospedales, 2015), Sketch-a-Net (77.95%) (Yu et al., 2017), SketchNet (80.42%) (Zhang et al., 2016), Thinning-DNN (74.3%) (Ahn, Shin & Kim, 2016), CNN-PCA-SVM (72.5%) (Sert & Boyacı, 2019), Hybrid-CNN (84.42%) (Zhang et al., 2020), and human recognition accuracy 73% (Eitz, Hays & Alexa, 2012). The effectiveness of the proposed Sketch-DeepNet architecture makes it an ideal candidate for sketch classification-related problems such as sketch recognition (Eitz, Hays & Alexa, 2012), sketch-based image retrieval (Eitz et al., 2010), and forensic sketch analysis (Klare, Li & Jain, 2010).

The remainder of the article is organized as follows. The “Related Work” section discusses the related work. The “Proposed Sketch-DeepNet Methodology” section presents the proposed CNN methodology for Sketch-DeepNet. In the section “Experiments”, the investigations are performed to validate the proposed model. In the section “Results and Evaluations”, the comparative analysis of the proposed model with the existing state-of-the-art models is carried out. Finally, in the section “Conclusion”, the proposed work is concluded, followed by the future work.

## Related works

In Sarvadevabhatla & Babu (2015), the authors implemented CNNs based architecture to classify hand-drawn sketches and object categories. The two famous CNN’s based models presented for hand-drawn sketches are—AlexNet CNN (Krizhevsky, Sutskever & Hinton, 2012) and the revised version of LeNet (LeCun et al., 1998). AlexNet and LeNet performed well as compared to the state-of-the-art approaches (Dalal & Triggs, 2005; Li et al., 2015). The first significant attempt on using (DCNNs) for free-hand-drawn sketch classification is Sketch-a-Net, which is first introduced in Yu et al. (2015) and then later on improved in Yu et al. (2017). The researchers made many changes in the latest network as compared to the earlier version of Sketch-a-Net. In an enhanced version (Yu et al., 2017), the authors used stroke timing and geometry information for data augmentation strategy. The dataset was deformed to increase the training set’s size and remove the problem of over-fitting. The classification accuracy achieved was 77.95% on the TU-Berlin sketch dataset. In another work (Zhang et al., 2016), DCNN based sketch classification called SketchNet was presented, but the main objective of SketchNet was to learn the shared structures which present between sketch and real images. SketchNet consists of three subnets: R-Net extracted features from the actual images, S-Net was used on the sketch images, and C-Net was used to discover the common structures between real images and sketches. For example, a sketch image of an object is used as an input and performed its initial category predictions on the pre-trained sketch model. Then the test pairs are constructed based on their visual similarity to achieve the prediction scores of each test image pair. SketchNet achieved the classification accuracy of 80.42% on the TU-Berlin sketch benchmark. A context-based self-learning algorithm that can learn sketches from a few samples of sketch images was presented in Yesilbek & Sezgin (2017). The experiments were carried out with the execution pipeline, consisting of four stages, *i.e*., candidate extraction, conservative rejection, self-learning, and performance measurement. The performance was not accurate enough to be used in a real-world environment. A multi-stage training methodology using SketchANet and AlexNet is presented in Bui et al. (2018). The strategy combines contrastive and triple regression loss functions to improve the algorithm’s performance. The primary aim was to develop a training strategy for partial sharing networks. The methodology was complex and challenging to implement in a real-world scenario. In Yang & Hospedales (2015), a new (DCNN) based architecture for sketch classification is presented. The architecture consists of six convolutional layers and two fully connected layers. After the first, second, and fifth convolutional layers, a top pooling layer of 3 × 3 was placed. The image size of 200 × 200 pixels was selected. The classification accuracy achieved was 72.2% on the TU-Berlin dataset. Another study on sketch classification is DeformSketchNet (Dai et al., 2017) which used deformable convolutions to augment spatial sampling locations in convolutions to learn robustness to geometric transformations. DeformSketchNet resized the images into 128 × 128-pixel files and applied random horizontal flips to augment the data for training. DeformSketchNet achieved 62.6% accuracy on the TUBerlin dataset. In another work (Ahn, Shin & Kim, 2016), the authors designed a deeper network to improve the performance and achieved 74.3% on the TU-Berlin dataset. Other models proposed for hand-drawn sketches recognitions are (LR), (SVMs), CNN, and transfer learning (Phu, Xiao & Muindi, 2018). After implementing these algorithms and analyzing their results, they concluded that a simplified CNN could balance accuracy and training time. The CNN-based models achieved 89.02%, 87.22%, and 77.46% accuracy on 3, 10, and 50 classes of QuickDraw (Google, 2017) dataset. The main drawback of their approach is the reduction in the accuracy when the number of classes is increasing.

In Zhou & Jia (2020), a sketch recognition learning methodology that focussed on visual geometry group 16 convolutional neural network (VGG16 CNN) is presented. The method is divided into two parts. The first part represents the pre-trained CNN-based feature extraction pipeline, and the other party extracts the contextual features. Initially, a graph is constructed and then traverses this graph according to the critical points of sketch strokes or the fixed numbers of steps. A contextual feature was obtained while spanning the length of the selected number of relationships. In the end, the joint Bayesian model was used to measure the relationship between multiple feature vectors. The final experimental result represents that VGG16 CNN achieved the classification accuracy of approx. 78% on the TU-Berlin dataset. The VGG16 CNN approach leads to some deficiencies in the training phase. It consumed more time in execution during extracting contextual features.

A transfer learning-based approach for sketch recognition is presented in Sert & Boyacı (2019). The method used a pre-trained model for feature extraction and fine-tuned it on the TU-Berlin dataset. The pre-trained model considered ResNet-50 (Singh & Kisku, 2018) which was trained on the MNIST dataset (LeCun et al., 1998). After choosing the pre-trained ResNet-50 model, a flatten layer was added, one drop-out layer to reduce over-fitting, and one fully connected layer for classification. After this, the model passed training data of 10,000 images through the network to fine-tune the model. A stochastic gradient optimizer, softmax activation, and cross-entropy were used to minimize the loss function. The model achieved a validation accuracy of 72.94%. A sketch recognition model based on (CNN) is presented in Kabakus (2020). The model was responsible for learning the stroke patterns of sketches and predicting the classes of given sketches. The model consisted of 21 layers, out of which four were convolutional layers, five batch normalization layers, two max-pooling layers, one flatten layer, five drop-out layers, and four fully connected layers. The rectified linear units (ReLU) was used as the activation function for all the activations except for the output layer. The categorical cross-entropy (Softmax Loss) was utilized as a loss function for the previous layer. The adaptive moment estimation (Adam) algorithm (Kingma & Ba, 2014), which is an extension of stochastic gradient descent (SGD), was exploited as the optimization algorithm of the model. The model achieved a classification accuracy of 89.53% on the QuickDraw dataset.

A graph neural network (GNN) based architecture is also applied on the free-hand sketch recognition (Xu, Joshi & Bresson, 2021). The author’s designed a multigraph transformer based on (GNN) for learning representations of sketches of multiple graphs. The GNN based model concurrently capture global and local geometric stroke structures as well as temporal information and achieved 89.45% classification accuracy on the Google QuickDraw dataset. In this connection, an end-to-end single branch network architecture RNN-Rasterization-CNN (Sketch-R2CNN) is presented to entirely leverage the vector format of sketches (Li et al., 2020). Sketch-R2CNN only takes a vector sketch as input and uses an RNN for feature extraction for each sketch point. A neural line rasterization (NLR) module was developed, which converts the vector sketch with the per-point features to multi-channel point feature maps differently. Afterward, an off-the-shelf CNN takes the resulting point feature maps and predicts the final object category as output. The performance of Sketch-R2CNN was evaluated using two sketch datasets. The first one was the TU-Berlin dataset (Eitz, Hays & Alexa, 2012) which contains 250 object categories, and the other one was the QuickDraw dataset which includes 345 categories of sketch objects. The Sketch-R2CNN achieved 85.4% of classification accuracy on the TU-Berlin dataset and 85.3% accuracy on the QuickDraw dataset.

## Proposed sketch-deepnet methodology

**Figure 1:**
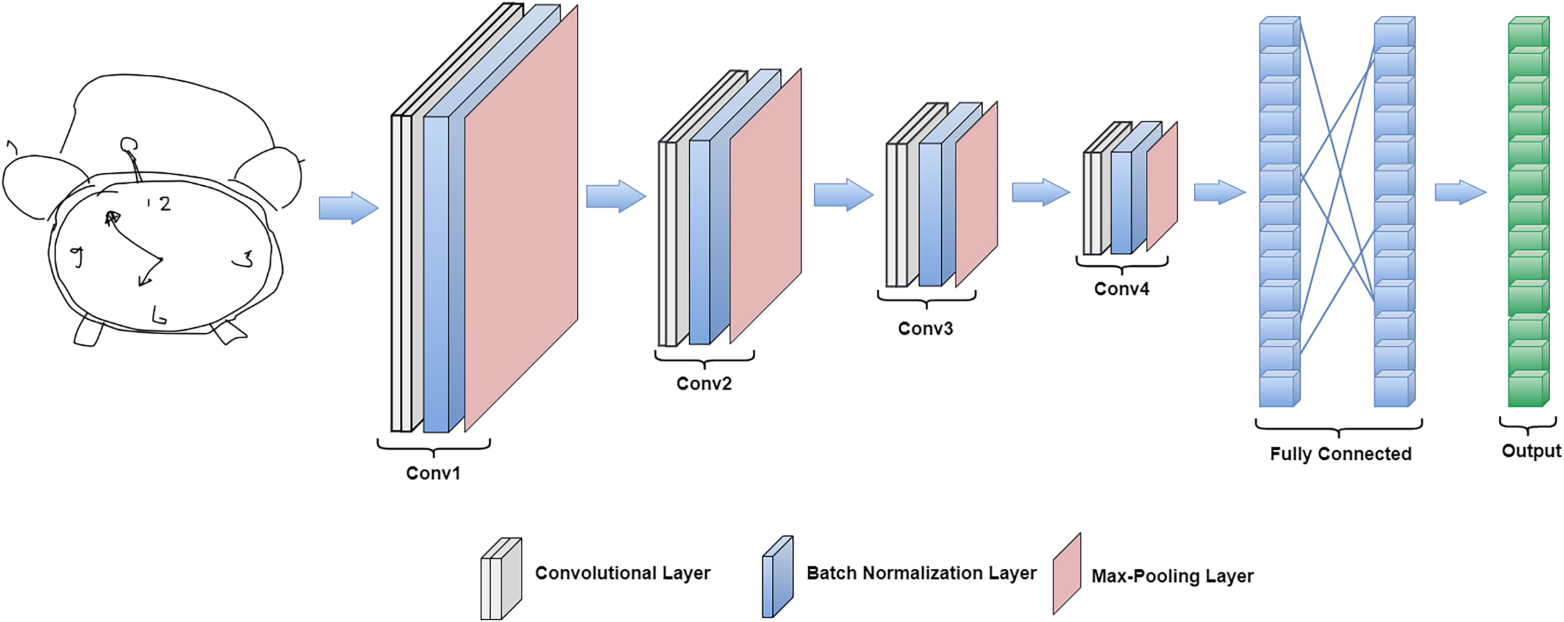
Illustration of our CNN model.

CNN is very popular in the field of computer vision because of its proven success in solving image classification problems (Krizhevsky, Sutskever & Hinton, 2012; Liu & Deng, 2015; Glorot, Bordes & Bengio, 2011). In literature, CNN is found immensely fine at learning local and global features from image data. The recognition performance of many image databases such as ImageNet, NORB, MNIST and CIFAR10 is improved by using CNN-based methodologies. Image objects such as human faces or handwritten digits are formed by combining local and global features, hence, the combination of simple local features like curves and edges builds more complex features such as corners and shapes. Recently, CNN has also been used in free-hand sketch classification (Dai et al., 2017; Yang & Hospedales, 2015; Yu et al., 2015, 2017; Zhang et al., 2016; Ahn, Shin & Kim, 2016; Sert & Boyacı, 2019; Zhang et al., 2020; Zhou & Jia, 2020; Li et al., 2020) and the accuracy was improved as compared to the classical machine learning algorithms (Dalal & Triggs, 2005, Li et al., 2015), but there is still room for improvement. Therefore, we design a new deep CNN architecture named Sketch-DeepNet for this purpose. The proposed deep CNN is described in Fig. 1.

### CNN architecture

The overall design of the proposed Sketch-DeepNet architecture is illustrated in Fig. 1. It is a seven-layer CNN architecture. The network input is a normalized sketch image with zero mean and unit variance. There are four convolutional layers in the proposed architecture. Batch normalization and max-pooling are applied after each convolutional layer. Then three linear (fully connected) layers are added. Drop-out regularisation of value 0.5 is applied after the second linear layer. The rectified linear unit (ReLU) 1 (Eq. (1)) (Dahl, Sainath & Hinton, 2013) activation function is used after each convolutional and linear layer except the final layer. The final layer has 250 output features representing 250 categories (number of classes in the TU-Berlin dataset). The detailed parameter setting of the proposed CNN architecture is shown in Table 1.

**Table 1:** The architecture of Sketch-DeepNet

Layers	Kernel	In-channels	Out-channels
Conv1	5 × 5	3	64
Relu	–	–	–
Maxpool	2 × 2	–	–
Conv2	3 × 3	64	128
Relu	–	–	–
Maxpool	2 × 2	–	–
Conv3	3 × 3	128	255
Relu	–	–	–
Maxpool	2 × 2	–	–
Conv4	3 × 3	255	512
Relu	–	–	–
Maxpool	2 × 2	–	–
FC1	–	512 × 2 × 2	1,024
Relu	–	–	–
FC2	–	1,024	1,024
Relu	–	–	–
Dropout (0.25)	–	–	–
Output	–	1,024	1,024

Rectified linear unit equation:

(1) $$f(x) = max(0,x)$$

### Convolution for feature extraction

Small image pixels are directly used as input to the networks to address the image classification problem. However, it is noted that even small image patches contain many pixels, resulting in a massive amount of weight parameters to be trained (Liu & Deng, 2015). According to the Vapnik–Chervonenkis (VC) dimension theory (Vapnik & Chervonenkis, 2015), the significant number of weight parameters ends in more complex systems that require numerous training samples to prevent the over-fitting problem. CNN models significantly simplify the learning process by integrating weights into a substantially smaller kernel size. Compared to conventional fully connected neural networks, CNN is more powerful and accelerated in extracting the features.

### Convolutional layers

In CNN, convolutional neuron layers are the main building blocks. Three two-dimensional channels are input to the first convolutional layer. Various two-dimensional channels are input to the second convolutional layer. The number of input channels can differ from the number of output channels (Li et al., 2014). A single output channel is calculated by using the equation given below:

(2) $${A_y} = f\left(\sum\limits_{x = 1}^N {{I_x}} *{K_{x,y}} + {B_y}\right)$$

${I_x}$ = input matrix

${K_x}{,_y}$ = kernel matrix

${B_y}$ = bias value

f = non-linear function

${A_y}$ = output matrix

Each particular set of kernel matrices indicates a feature extractor that extracts features from input channels. The learning procedure aims to determine kernel matrices that extract reliable and robust features to classify sketch images. The connection of neurons in a neural network is optimized by the back-propagation process, where connection weights train the kernel matrices.

### Pooling layers

**Figure 2:**
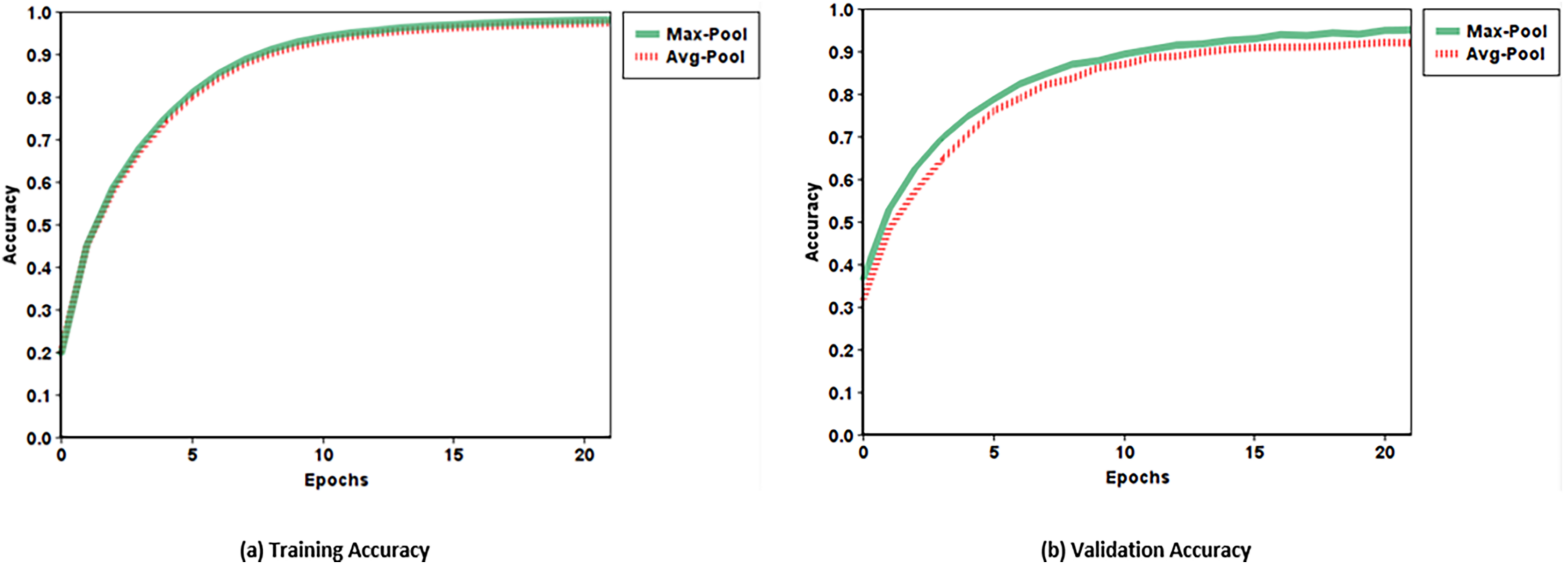
Training and validation accuracy of max-pooling and average-pooling.

In CNN, the pooling layer plays a vital role in the reduction of feature dimension. A pooling algorithm can be used to merge the adjacent elements in the output channels of convolution. Pooling algorithms include min-pooling, average-pooling, and max-pooling. The proposed network uses the max-pooling with kernel size 2 × 2, which takes the maximum value from the four adjacent elements of the input channel to create a single component in the output channel. In the error back-propagation process, the gradient indication is passed back to the neurons, contributing to the pooling output. The model achieves validation accuracy of 95.05% by using max-pooling which is 4% higher than using average-pooling. The performance of using max-pooling and average-pooling is also shown in Fig. 2.

### Activation function

**Figure 3:**
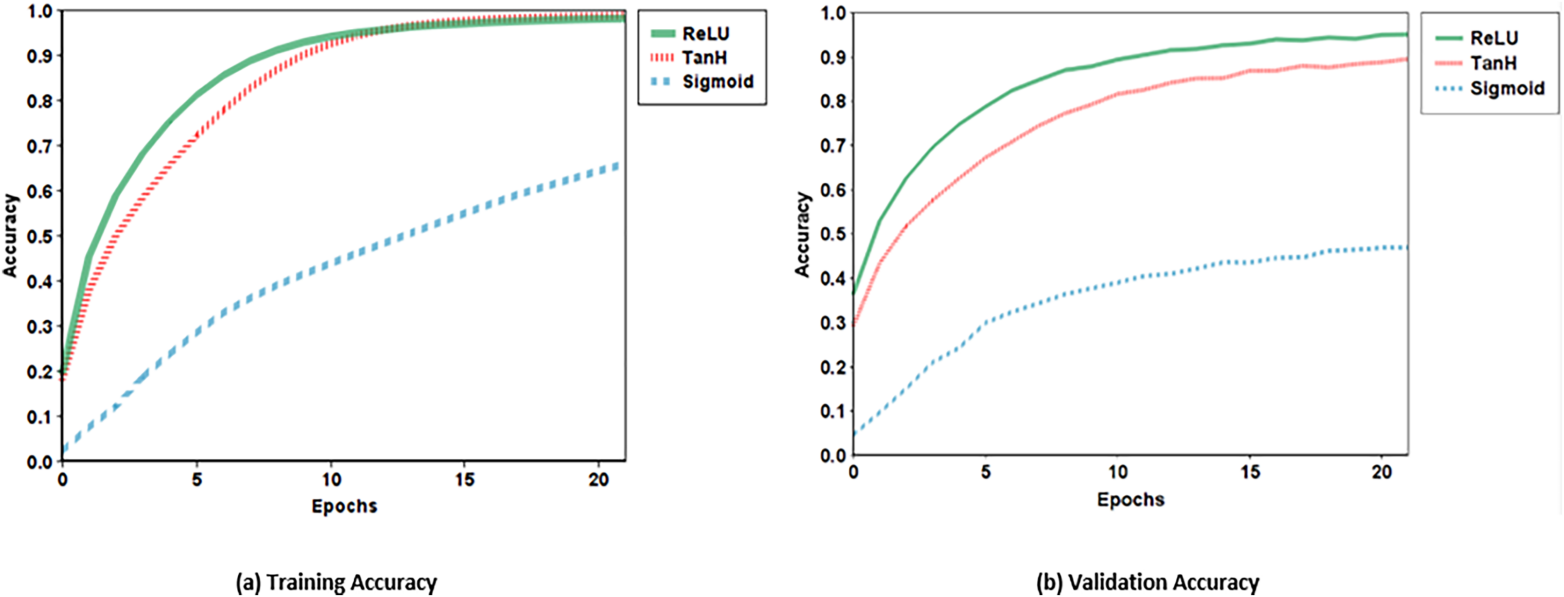
Training and validation accuracy of ReLU, Sigmoid and TanH.

Non-linear functions are used as the activation function for neurons in artificial neural networks *e.g*., the hyperbolic tangent function (Eq. (3)) (Glorot, Bordes & Bengio, 2011), and the sigmoid function (Eq. (4)) (Glorot, Bordes & Bengio, 2011), both are most commonly used saturating non-linear activation functions. In saturating non-linear activation functions, when input increases, the output gradient decreases and approaches zero. The classification performance and the learning speed of CNN can be improved using non-saturating non-linear functions like rectified linear unit (ReLU) (Li et al., 2014). In the proposed CNN model, the ReLU activation function is implemented. The outcomes indicated that the performance is improved than using any saturating non-linear function such as Sigmoid and TanH. The training and validation accuracies for ReLU, Sigmoid, and TanH are shown in Fig. 3.

TanH function equation:

(3) $$f(x) = tanh(x)$$

Sigmoid function equation:

(4) $$f(x) = 1/(1 + {e^{ - x}})$$

### Drop-out layer

Drop-out is a regularisation technique and it is used to avoid neural network from over-fitting. During training of the neural network, the dropout actually works like creating a narrow network with unique combinations of neurons in dense or fully connected layers and randomly dropping these combinations at different points. In every training iteration, a new narrow network is created with different neurons randomly dropped based on the probability of hyper-parameter *p*. Training the neural network by using dropout is like training various different narrow networks and then combining them to create a single neural network which holds the properties of each narrow network. In Dahl, Sainath & Hinton (2013) the performance of the neural network is improved by applying a drop-out algorithm. In the proposed methodology, a dropout algorithm is used and the hyper-parameter’s probability *p* is selected to 0.5.

## Experiments

### Dataset

**Figure 4:**
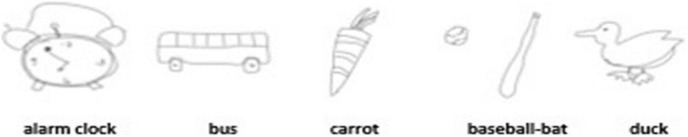
Sample sketches from the TU-Berlin dataset.

The proposed Sketch-DeepNet model used the TU-Berlin dataset collected from 1,350 participants from the crowd-sourcing platform (Amazon Mechanical Turk). This dataset consists of 20,000 sketches across 250 categories. Sketches images are given as 1,111 × 1,111 pixels PNG files. The images are resized to 64 × 64 pixels because large size is unnecessary, particularly on sketch datasets. It is possible to use smaller sizes and get better results. As sketches are black and white images, each pixel value is converted to the range [0, 1]. The dataset is divided into three sub-datasets 70/20/10 as training/validation/test. The sample sketches from TU-Berlin dataset are shown in Fig. 4

In the pre-processing stage, the images are resized into 64 × 64 pixels and apply a random vertical and horizontal flip to the images to augment the dataset. After using augmentation techniques, the size of the dataset increase by a factor of 30 and becomes 500,000 images. Then the dataset is randomly split into train, validation, and test sets.

### Implementation details

**Figure 5:**
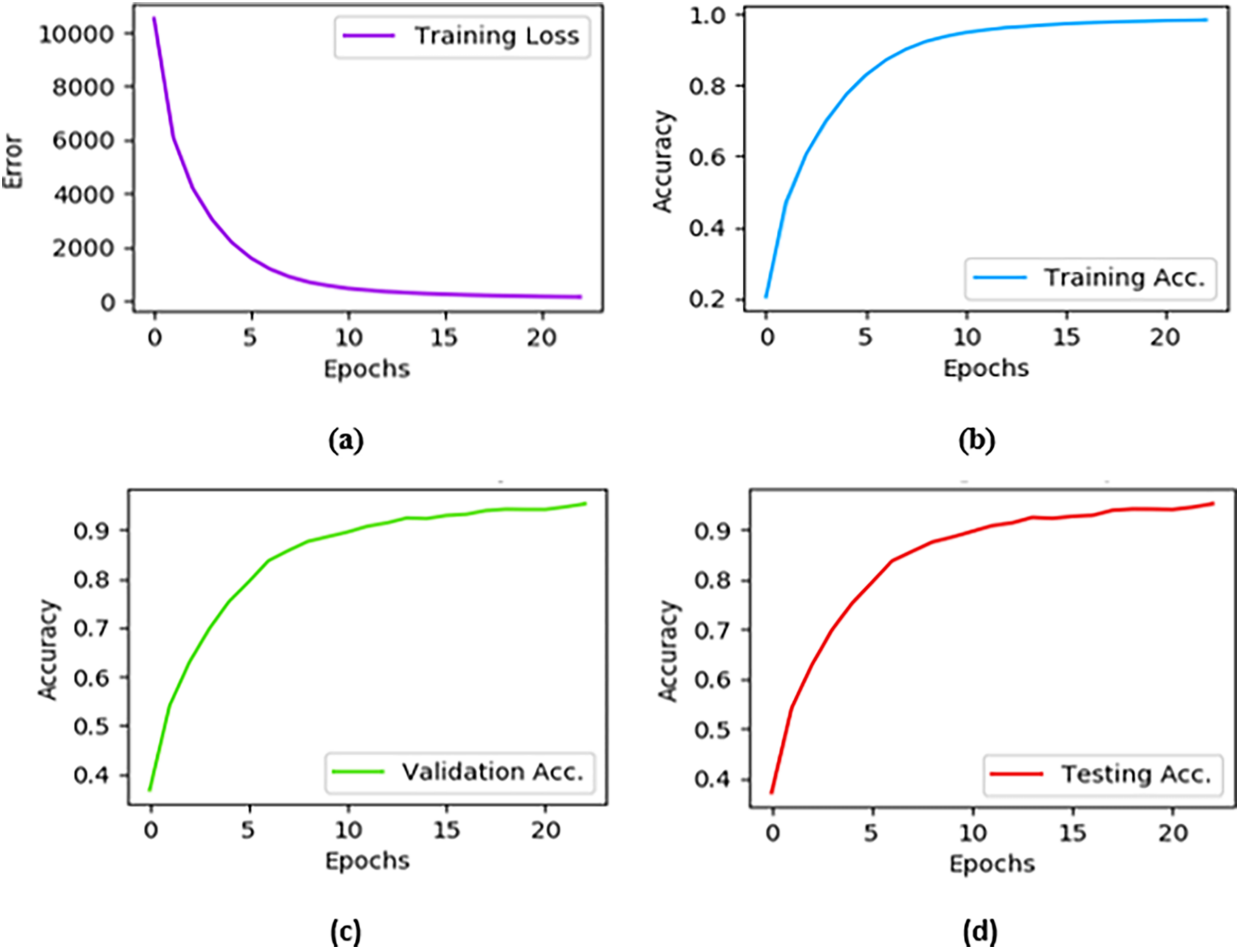
Training loss represents the error on the training set of data. In graph (A) the training loss value continuously decreases and it almost reaches zero at 22 epoch. (B) The training accuracy curve shows model learning and the learning stopped at the point where the model converges to prevent over-learning. The validation accuracy plot in (C) represents the generalization ability of the model and the model achieved 94% accuracy on the validation set. The testing accuracy (D) depicts the performance of the model on test data or unseen data. The tests are attempted after completion of every epoch and the final result recorded 95%.

A flexible neural computing software platform is required to implement the proposed Sketch-DeepNet CNN architecture. Therefore, we selected the open-source neural network framework Pytorch to train the proposed network. After hyper parameters tuning, the learning rate and batch size are set to 0.01 and 128, respectively. There are approximately 2,735 total batches. The network trained up to 22 epochs because it became stable after this point. The network is trained on NVIDIA Tesla P100 GPU with 16 GB memory. GPU is used to accelerate the learning process because of the larger dataset and number of parameters. The system consists of 32 GB of RAM, 1TB of SSD, and an Intel i7 processor. The training loss, training accuracy, validation accuracy, and testing accuracy results of the proposed Sketch-DeepNet are shown in Fig. 5.

## Results and evaluations

**Figure 6:**
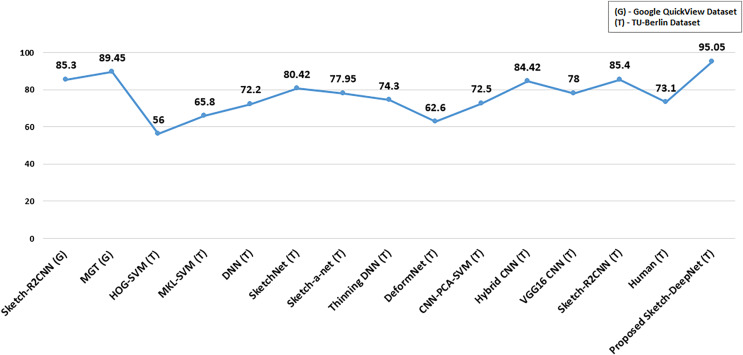
Comparison with state-of-the-art results on sketch recognition.

The proposed Sketch-DeepNet is compared with existing state-of-the-art standard techniques (Dalal & Triggs, 2005; Li et al., 2015; Yang & Hospedales, 2015; Zhang et al., 2016; Yu et al., 2017; Ahn, Shin & Kim, 2016; Dai et al., 2017; Sert & Boyacı, 2019; Zhang et al., 2020; Zhou & Jia, 2020; Li et al., 2020) as shown in Fig. 6. These techniques can be divided into two categories based on the features they utilize to represent sketches. One is hand-engineered features, and the other is deep learning-based features. Handcrafted features-based techniques are HOGSVM (Dalal & Triggs, 2005) and MKL-SVM (Li et al., 2015) with the classification accuracy of 56% and 65.8% respectively. The deep learning-based techniques are DeformNet (Dai et al., 2017) with a classification accuracy of 62.6%, SketchNet (Zhang et al., 2016) having classification accuracy of 80.42%, Thinning-DNN (Ahn, Shin & Kim, 2016) having classification accuracy of 74.3%, CNN-PCA-SVM (Sert & Boyacı, 2019) and its classification accuracy was 72.5%, Hybrid-CNN (Zhang et al., 2020) having classification accuracy of 84.42%, VGG16-CNN (Zhou & Jia, 2020) with the classification accuracy of approx. 78%, Sketch-R2CNN (Li et al., 2020) having classification accuracy of 85.4%, Sketch-a-Net (Yu et al., 2017) and its resultant classification accuracy was 77.95%, and finally, the Sketch-DNN (Yang & Hospedales, 2015) having the accuracy of 72.2%. The proposed model accuracy is 95.05%. So, it is clear from the comparisons of results that the proposed model performs well as compared to the existing models (Dalal & Triggs, 2005; Dai et al., 2017; Yang & Hospedales, 2015; Yu et al., 2017; Zhang et al., 2016; Ahn, Shin & Kim, 2016; Sert & Boyacı, 2019; Zhang et al., 2020; Li et al., 2015; Zhou & Jia, 2020; Li et al., 2020). The following observation is also made:

1. The proposed Sketch-DeepNet remarkably outperforms all the existing methods (Dalal & Triggs, 2005; Dai et al., 2017; Yang & Hospedales, 2015; Yu et al., 2017; Zhang et al., 2016; Ahn, Shin & Kim, 2016; Sert & Boyacı, 2019; Zhang et al., 2020; Li et al., 2015; Zhou & Jia, 2020; Li et al., 2020) designed for sketch recognition.
2. The 95.05% classification accuracy of the proposed Sketch-DeepNet model also outperforms the human-computer interaction having a classification accuracy of 73% (Eitz, Hays & Alexa, 2012).
3. Upon close examination at the category level, we concluded that the proposed Sketch-DeepNet performed well at fine-grained object categories. This performance shows that Sketch-DeepNet learned more discriminative feature representation than hand-crafted features.
4. The proposed model also performs well on geometric invariance property. Using data augmentation techniques (horizontal and vertical flips), we trained the model on randomly rotated sketch images that overcome the geometry invariance problem.
5. Resizing sketch images to 64 × 64 not only reduces the training time but also benefits learning accuracy. Even in small sizes, sketches are powerful in representing information, and it’s more convenient to work with small-sized input for CNN.
6. The proposed model will fall short if any ambiguous sketch is provided as an input to the model or if the input sketch does not belong to the categories present in the TU-Berlin dataset, *e.g*., if we feed the model with the sketch image of a digital wrist-watch but the model is trained on an analogue wrist-watch, then the model’s accuracy for the wrist-watch category will be affected.

**Figure 7:**
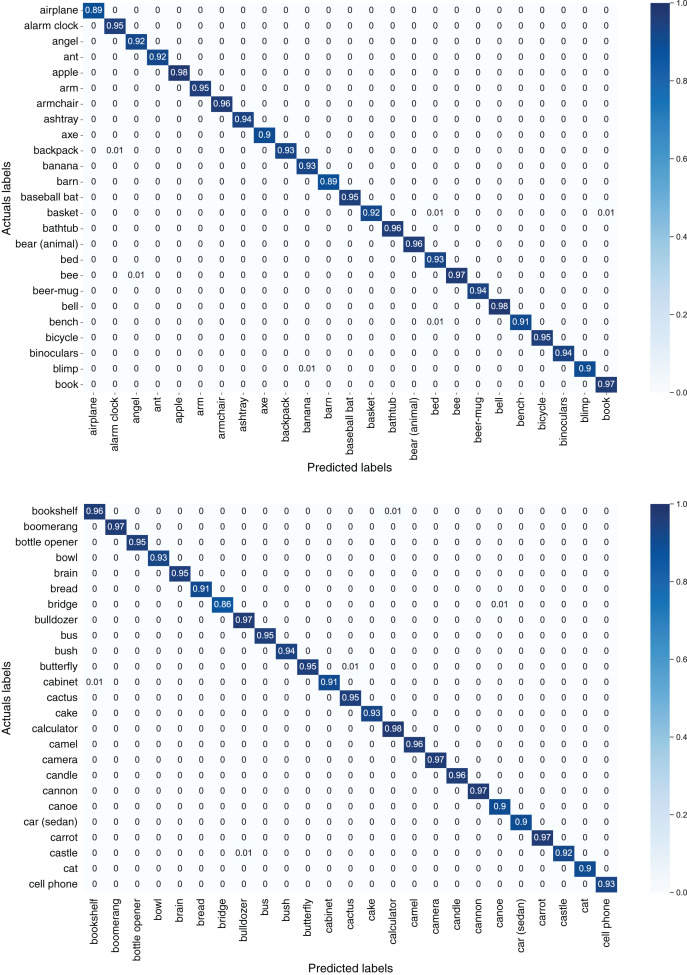
Confusion matrix of first 50 classes of the TU-Berlin dataset.

**Figure 8:**
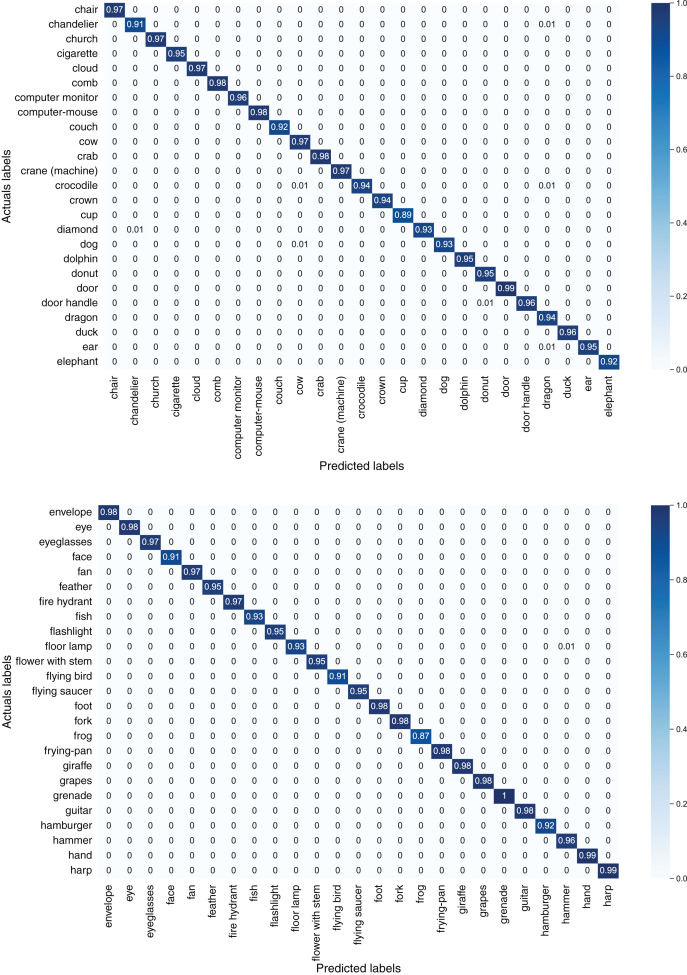
Confusion matrix of second 50 classes of the TU-Berlin dataset.

**Figure 9:**
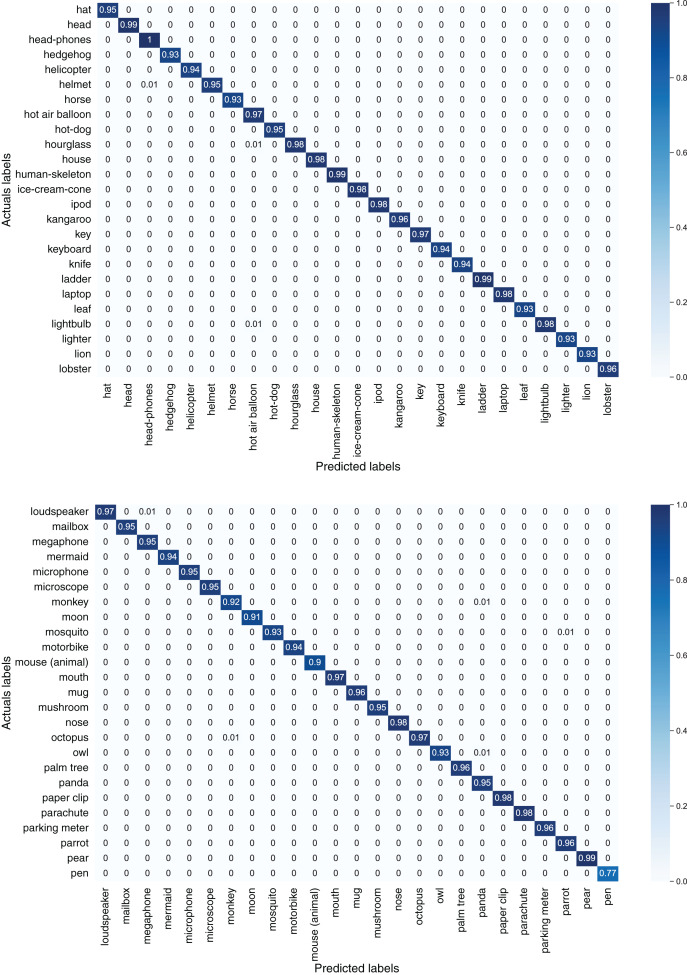
Confusion matrix of third 50 classes of the TU-Berlin dataset.

**Figure 10:**
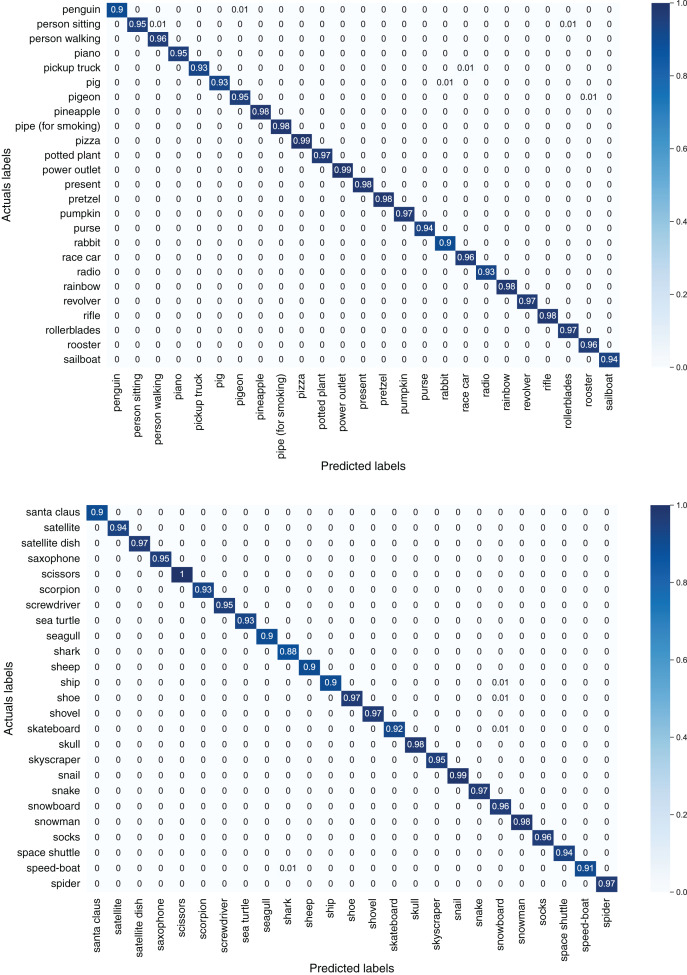
Confusion matrix of forth 50 classes of the TU-Berlin dataset.

**Figure 11:**
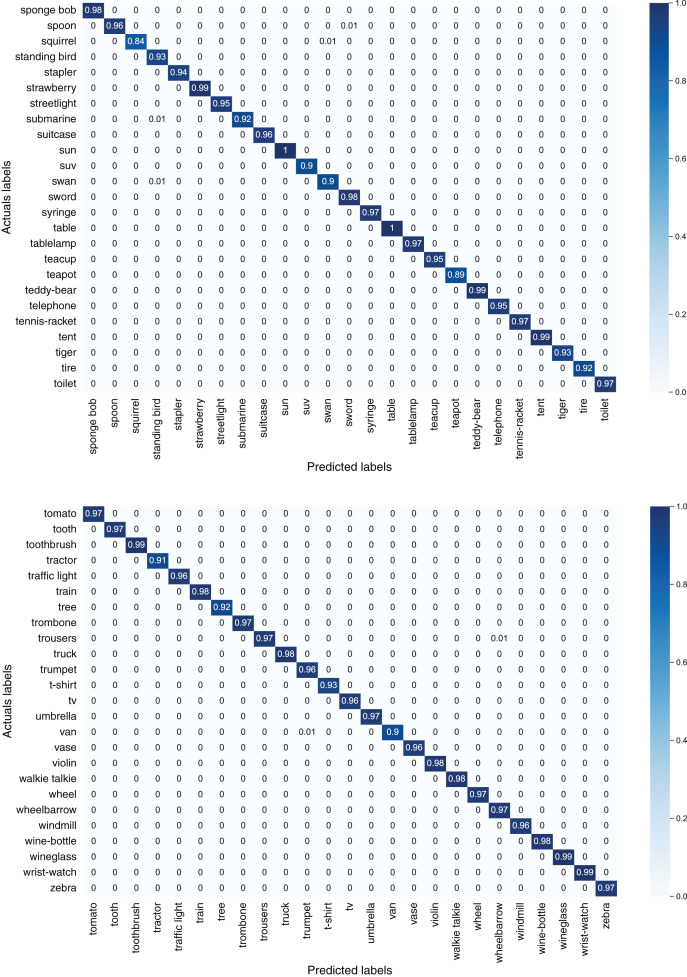
Confusion matrix of fifth 50 classes of the TU-Berlin dataset.

Figure 7 depicts confusion matrices of first 50 classes of the TU-Berlin dataset, Fig. 8 depicts confusion matrices of second 50 classes, Fig. 9 shows confusion matrices of third 50 classes, Fig. 10 shows the confusion matrices of fourth 50 classes, and Fig. 11 represents confusion matrices of the remaining 50 classes of the TU-Berlin dataset.

**Figure 12:**
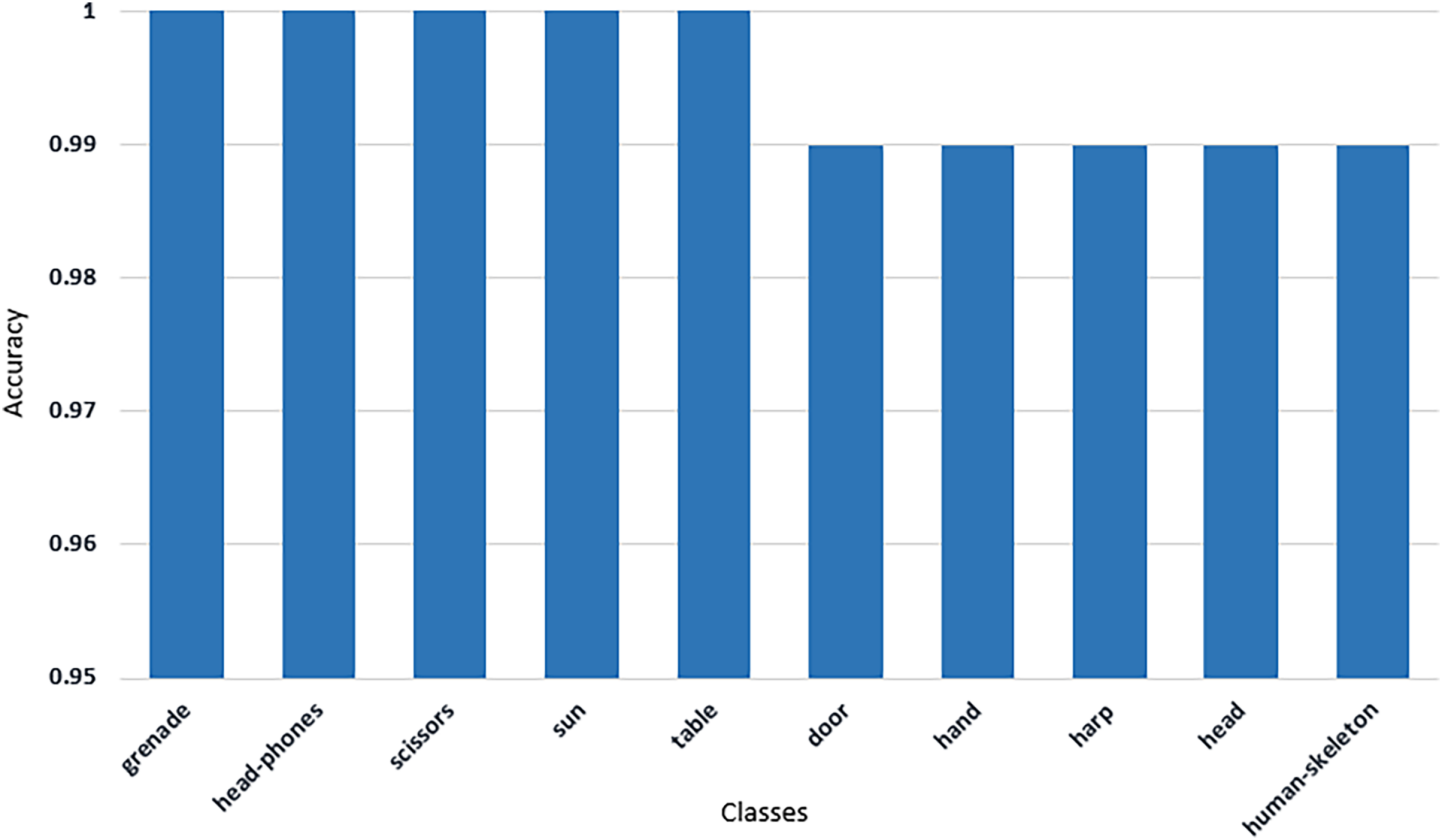
Top 10 classes with maximum classification accuracy achieved by Sketch-DeepNet.

**Figure 13:**
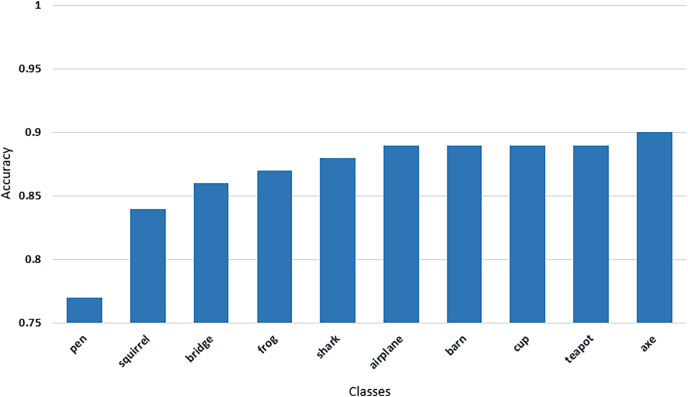
The worst 10 classes with minimum classification accuracy achieved by Sketch-DeepNet.

From Figs. 7–11 we can see that some classes are 100% correctly classified using proposed Sketch-DeepNet i-e grenade, head-phones and scissors, *etc*., while some other classes pen, squirrel and bridge are correctly classified with the accuracy of 77%, 84% and 86% respectively. Figure 12 depicts the top ten classes for which the proposed model Sketch-DeepNet achieved the highest classification accuracy, whereas Fig. 13 depicts the ten worse classes with the lowest classification accuracy.

## Conclusion

This article focused on free-hand sketch classification. Free-hand sketch classification is the first and most critical task to be considered while designing sketch-related AIoT systems, such as a sketch context recognition system or a sketch-based searching system. To accomplish this task, this article proposed a deep neural network-based free-hand sketch recognition model called Sketch-DeepNet. Sketch-DeepNet achieved an efficient representation of sketches and increased the accuracy of sketch recognition to 95.05% as compared to the existing state-of-the-art techniques DeformNet (62.6%) (Dai et al., 2017), Sketch-DNN (72.2%) (Yang & Hospedales, 2015), Sketch-a-net (77.95%) (Yu et al., 2017), SketchNet (80.42%) (Zhang et al., 2016), Thinning-DNN (74.3%) (Ahn, Shin & Kim, 2016), CNN-PCA-SVM (72.5%) (Sert & Boyacı, 2019), Hybrid-CNN (84.42%) (Zhang et al., 2020), VGG-CNN (approx. 78%) (Zhou & Jia, 2020), Sketch-R2CNN (85.4%) (Li et al., 2020), and human recognition accuracy 73% (Eitz, Hays & Alexa, 2012). The proposed Sketch-DeepNet model used the TU-Berlin dataset (Eitz, Hays & Alexa, 2012). The efficiency of the proposed model makes it a perfect candidate for sketch classification-related problems such as sketch recognition (Eitz, Hays & Alexa, 2012), sketch-based image retrieval (Eitz et al., 2010), and forensic sketch analysis (Klare, Li & Jain, 2010). The article also concluded that the proposed model is independent of the geometric invariance property of sketch images and small image size profits in training time and learning accuracy. The learned sketch feature representation is a great advantage for sketch-related applications such as automatic sketch synthesis and sketch-based image retrieval.

In the future, we would further flourish our most optimistic approach by conducting more extensive experiments. We will also explore the benefit of late fusion schemes. We would also love to explore utilizing transfer learning as a feature extractor.

## Supplemental Information

10.7717/peerj-cs.1186/supp-1 Supplemental Information 1 Research Code.

10.7717/peerj-cs.1186/supp-2 Supplemental Information 2 Dataset used for training, validation and testing.

## Additional Information and Declarations

### Data Availability

The following information was supplied regarding data availability: The raw data and code are available in the Supplemental Files.
