# Supplementary figures and images for: Context awareness based Sketch-DeepNet architecture for hand-drawn sketches classification and recognition in AIoT (part 2 of 2)
Source: PeerJ Comput Sci. 2023 Apr 27;9:e1186. doi: 10.7717/peerj-cs.1186 (PMC10280188; doi:10.7717/peerj-cs.1186)

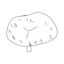

Supplement: Supplemental Information 2 [file peerj-cs-09-1186-s002.zip › Dataset/alarm clock/120.png]

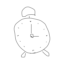

Supplement: Supplemental Information 2 [file peerj-cs-09-1186-s002.zip › Dataset/alarm clock/121.png]

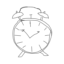

Supplement: Supplemental Information 2 [file peerj-cs-09-1186-s002.zip › Dataset/alarm clock/122.png]

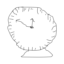

Supplement: Supplemental Information 2 [file peerj-cs-09-1186-s002.zip › Dataset/alarm clock/123.png]

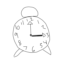

Supplement: Supplemental Information 2 [file peerj-cs-09-1186-s002.zip › Dataset/alarm clock/124.png]

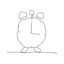

Supplement: Supplemental Information 2 [file peerj-cs-09-1186-s002.zip › Dataset/alarm clock/125.png]

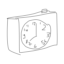

Supplement: Supplemental Information 2 [file peerj-cs-09-1186-s002.zip › Dataset/alarm clock/126.png]

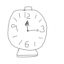

Supplement: Supplemental Information 2 [file peerj-cs-09-1186-s002.zip › Dataset/alarm clock/127.png]

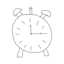

Supplement: Supplemental Information 2 [file peerj-cs-09-1186-s002.zip › Dataset/alarm clock/128.png]

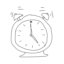

Supplement: Supplemental Information 2 [file peerj-cs-09-1186-s002.zip › Dataset/alarm clock/129.png]

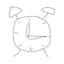

Supplement: Supplemental Information 2 [file peerj-cs-09-1186-s002.zip › Dataset/alarm clock/130.png]

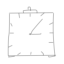

Supplement: Supplemental Information 2 [file peerj-cs-09-1186-s002.zip › Dataset/alarm clock/131.png]

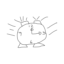

Supplement: Supplemental Information 2 [file peerj-cs-09-1186-s002.zip › Dataset/alarm clock/132.png]

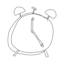

Supplement: Supplemental Information 2 [file peerj-cs-09-1186-s002.zip › Dataset/alarm clock/133.png]

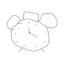

Supplement: Supplemental Information 2 [file peerj-cs-09-1186-s002.zip › Dataset/alarm clock/134.png]

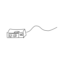

Supplement: Supplemental Information 2 [file peerj-cs-09-1186-s002.zip › Dataset/alarm clock/135.png]

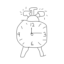

Supplement: Supplemental Information 2 [file peerj-cs-09-1186-s002.zip › Dataset/alarm clock/136.png]

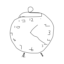

Supplement: Supplemental Information 2 [file peerj-cs-09-1186-s002.zip › Dataset/alarm clock/137.png]

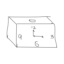

Supplement: Supplemental Information 2 [file peerj-cs-09-1186-s002.zip › Dataset/alarm clock/138.png]

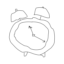

Supplement: Supplemental Information 2 [file peerj-cs-09-1186-s002.zip › Dataset/alarm clock/139.png]

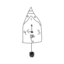

Supplement: Supplemental Information 2 [file peerj-cs-09-1186-s002.zip › Dataset/alarm clock/140.png]

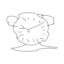

Supplement: Supplemental Information 2 [file peerj-cs-09-1186-s002.zip › Dataset/alarm clock/141.png]

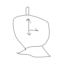

Supplement: Supplemental Information 2 [file peerj-cs-09-1186-s002.zip › Dataset/alarm clock/142.png]

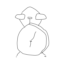

Supplement: Supplemental Information 2 [file peerj-cs-09-1186-s002.zip › Dataset/alarm clock/143.png]

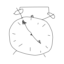

Supplement: Supplemental Information 2 [file peerj-cs-09-1186-s002.zip › Dataset/alarm clock/144.png]

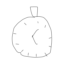

Supplement: Supplemental Information 2 [file peerj-cs-09-1186-s002.zip › Dataset/alarm clock/145.png]

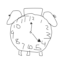

Supplement: Supplemental Information 2 [file peerj-cs-09-1186-s002.zip › Dataset/alarm clock/146.png]

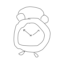

Supplement: Supplemental Information 2 [file peerj-cs-09-1186-s002.zip › Dataset/alarm clock/147.png]

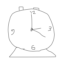

Supplement: Supplemental Information 2 [file peerj-cs-09-1186-s002.zip › Dataset/alarm clock/148.png]

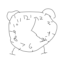

Supplement: Supplemental Information 2 [file peerj-cs-09-1186-s002.zip › Dataset/alarm clock/149.png]

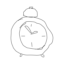

Supplement: Supplemental Information 2 [file peerj-cs-09-1186-s002.zip › Dataset/alarm clock/150.png]

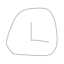

Supplement: Supplemental Information 2 [file peerj-cs-09-1186-s002.zip › Dataset/alarm clock/151.png]

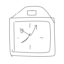

Supplement: Supplemental Information 2 [file peerj-cs-09-1186-s002.zip › Dataset/alarm clock/152.png]

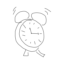

Supplement: Supplemental Information 2 [file peerj-cs-09-1186-s002.zip › Dataset/alarm clock/153.png]

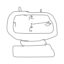

Supplement: Supplemental Information 2 [file peerj-cs-09-1186-s002.zip › Dataset/alarm clock/154.png]

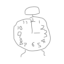

Supplement: Supplemental Information 2 [file peerj-cs-09-1186-s002.zip › Dataset/alarm clock/155.png]

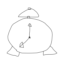

Supplement: Supplemental Information 2 [file peerj-cs-09-1186-s002.zip › Dataset/alarm clock/156.png]

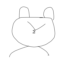

Supplement: Supplemental Information 2 [file peerj-cs-09-1186-s002.zip › Dataset/alarm clock/157.png]

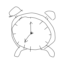

Supplement: Supplemental Information 2 [file peerj-cs-09-1186-s002.zip › Dataset/alarm clock/158.png]

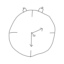

Supplement: Supplemental Information 2 [file peerj-cs-09-1186-s002.zip › Dataset/alarm clock/159.png]

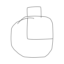

Supplement: Supplemental Information 2 [file peerj-cs-09-1186-s002.zip › Dataset/alarm clock/160.png]

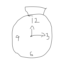

Supplement: Supplemental Information 2 [file peerj-cs-09-1186-s002.zip › Dataset/alarm clock/81.png]

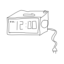

Supplement: Supplemental Information 2 [file peerj-cs-09-1186-s002.zip › Dataset/alarm clock/82.png]

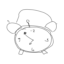

Supplement: Supplemental Information 2 [file peerj-cs-09-1186-s002.zip › Dataset/alarm clock/83.png]

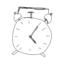

Supplement: Supplemental Information 2 [file peerj-cs-09-1186-s002.zip › Dataset/alarm clock/84.png]

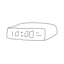

Supplement: Supplemental Information 2 [file peerj-cs-09-1186-s002.zip › Dataset/alarm clock/85.png]

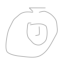

Supplement: Supplemental Information 2 [file peerj-cs-09-1186-s002.zip › Dataset/alarm clock/86.png]

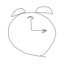

Supplement: Supplemental Information 2 [file peerj-cs-09-1186-s002.zip › Dataset/alarm clock/87.png]

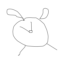

Supplement: Supplemental Information 2 [file peerj-cs-09-1186-s002.zip › Dataset/alarm clock/88.png]

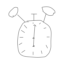

Supplement: Supplemental Information 2 [file peerj-cs-09-1186-s002.zip › Dataset/alarm clock/89.png]

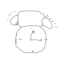

Supplement: Supplemental Information 2 [file peerj-cs-09-1186-s002.zip › Dataset/alarm clock/90.png]

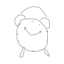

Supplement: Supplemental Information 2 [file peerj-cs-09-1186-s002.zip › Dataset/alarm clock/91.png]

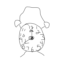

Supplement: Supplemental Information 2 [file peerj-cs-09-1186-s002.zip › Dataset/alarm clock/92.png]

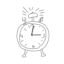

Supplement: Supplemental Information 2 [file peerj-cs-09-1186-s002.zip › Dataset/alarm clock/93.png]

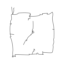

Supplement: Supplemental Information 2 [file peerj-cs-09-1186-s002.zip › Dataset/alarm clock/94.png]

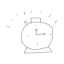

Supplement: Supplemental Information 2 [file peerj-cs-09-1186-s002.zip › Dataset/alarm clock/95.png]

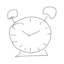

Supplement: Supplemental Information 2 [file peerj-cs-09-1186-s002.zip › Dataset/alarm clock/96.png]

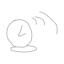

Supplement: Supplemental Information 2 [file peerj-cs-09-1186-s002.zip › Dataset/alarm clock/97.png]

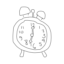

Supplement: Supplemental Information 2 [file peerj-cs-09-1186-s002.zip › Dataset/alarm clock/98.png]

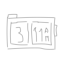

Supplement: Supplemental Information 2 [file peerj-cs-09-1186-s002.zip › Dataset/alarm clock/99.png]

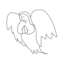

Supplement: Supplemental Information 2 [file peerj-cs-09-1186-s002.zip › Dataset/angel/161.png]

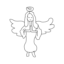

Supplement: Supplemental Information 2 [file peerj-cs-09-1186-s002.zip › Dataset/angel/162.png]

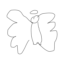

Supplement: Supplemental Information 2 [file peerj-cs-09-1186-s002.zip › Dataset/angel/163.png]

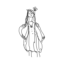

Supplement: Supplemental Information 2 [file peerj-cs-09-1186-s002.zip › Dataset/angel/164.png]

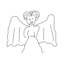

Supplement: Supplemental Information 2 [file peerj-cs-09-1186-s002.zip › Dataset/angel/165.png]

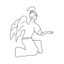

Supplement: Supplemental Information 2 [file peerj-cs-09-1186-s002.zip › Dataset/angel/166.png]

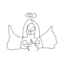

Supplement: Supplemental Information 2 [file peerj-cs-09-1186-s002.zip › Dataset/angel/167.png]

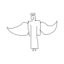

Supplement: Supplemental Information 2 [file peerj-cs-09-1186-s002.zip › Dataset/angel/168.png]

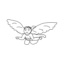

Supplement: Supplemental Information 2 [file peerj-cs-09-1186-s002.zip › Dataset/angel/169.png]

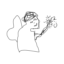

Supplement: Supplemental Information 2 [file peerj-cs-09-1186-s002.zip › Dataset/angel/170.png]

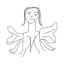

Supplement: Supplemental Information 2 [file peerj-cs-09-1186-s002.zip › Dataset/angel/171.png]

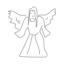

Supplement: Supplemental Information 2 [file peerj-cs-09-1186-s002.zip › Dataset/angel/172.png]

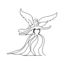

Supplement: Supplemental Information 2 [file peerj-cs-09-1186-s002.zip › Dataset/angel/173.png]

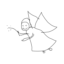

Supplement: Supplemental Information 2 [file peerj-cs-09-1186-s002.zip › Dataset/angel/174.png]

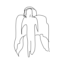

Supplement: Supplemental Information 2 [file peerj-cs-09-1186-s002.zip › Dataset/angel/175.png]

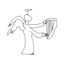

Supplement: Supplemental Information 2 [file peerj-cs-09-1186-s002.zip › Dataset/angel/176.png]

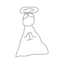

Supplement: Supplemental Information 2 [file peerj-cs-09-1186-s002.zip › Dataset/angel/177.png]

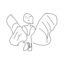

Supplement: Supplemental Information 2 [file peerj-cs-09-1186-s002.zip › Dataset/angel/178.png]

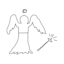

Supplement: Supplemental Information 2 [file peerj-cs-09-1186-s002.zip › Dataset/angel/179.png]

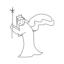

Supplement: Supplemental Information 2 [file peerj-cs-09-1186-s002.zip › Dataset/angel/180.png]

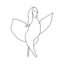

Supplement: Supplemental Information 2 [file peerj-cs-09-1186-s002.zip › Dataset/angel/181.png]

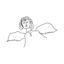

Supplement: Supplemental Information 2 [file peerj-cs-09-1186-s002.zip › Dataset/angel/182.png]

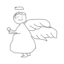

Supplement: Supplemental Information 2 [file peerj-cs-09-1186-s002.zip › Dataset/angel/183.png]

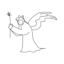

Supplement: Supplemental Information 2 [file peerj-cs-09-1186-s002.zip › Dataset/angel/184.png]

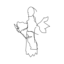

Supplement: Supplemental Information 2 [file peerj-cs-09-1186-s002.zip › Dataset/angel/185.png]

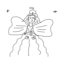

Supplement: Supplemental Information 2 [file peerj-cs-09-1186-s002.zip › Dataset/angel/186.png]

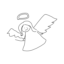

Supplement: Supplemental Information 2 [file peerj-cs-09-1186-s002.zip › Dataset/angel/187.png]

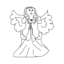

Supplement: Supplemental Information 2 [file peerj-cs-09-1186-s002.zip › Dataset/angel/188.png]

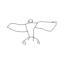

Supplement: Supplemental Information 2 [file peerj-cs-09-1186-s002.zip › Dataset/angel/189.png]

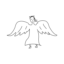

Supplement: Supplemental Information 2 [file peerj-cs-09-1186-s002.zip › Dataset/angel/190.png]

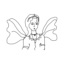

Supplement: Supplemental Information 2 [file peerj-cs-09-1186-s002.zip › Dataset/angel/191.png]

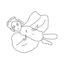

Supplement: Supplemental Information 2 [file peerj-cs-09-1186-s002.zip › Dataset/angel/192.png]

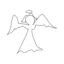

Supplement: Supplemental Information 2 [file peerj-cs-09-1186-s002.zip › Dataset/angel/193.png]

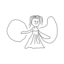

Supplement: Supplemental Information 2 [file peerj-cs-09-1186-s002.zip › Dataset/angel/194.png]

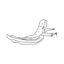

Supplement: Supplemental Information 2 [file peerj-cs-09-1186-s002.zip › Dataset/angel/195.png]

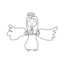

Supplement: Supplemental Information 2 [file peerj-cs-09-1186-s002.zip › Dataset/angel/196.png]

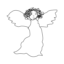

Supplement: Supplemental Information 2 [file peerj-cs-09-1186-s002.zip › Dataset/angel/197.png]

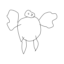

Supplement: Supplemental Information 2 [file peerj-cs-09-1186-s002.zip › Dataset/angel/198.png]

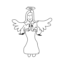

Supplement: Supplemental Information 2 [file peerj-cs-09-1186-s002.zip › Dataset/angel/199.png]

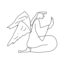

Supplement: Supplemental Information 2 [file peerj-cs-09-1186-s002.zip › Dataset/angel/200.png]
