# Supplementary figures and images for: Context awareness based Sketch-DeepNet architecture for hand-drawn sketches classification and recognition in AIoT (part 1 of 2)
Source: PeerJ Comput Sci. 2023 Apr 27;9:e1186. doi: 10.7717/peerj-cs.1186 (PMC10280188; doi:10.7717/peerj-cs.1186)

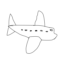

Supplement: Supplemental Information 2 [file peerj-cs-09-1186-s002.zip › Dataset/airplane/1.png]

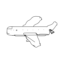

Supplement: Supplemental Information 2 [file peerj-cs-09-1186-s002.zip › Dataset/airplane/10.png]

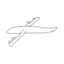

Supplement: Supplemental Information 2 [file peerj-cs-09-1186-s002.zip › Dataset/airplane/11.png]

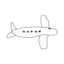

Supplement: Supplemental Information 2 [file peerj-cs-09-1186-s002.zip › Dataset/airplane/12.png]

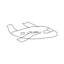

Supplement: Supplemental Information 2 [file peerj-cs-09-1186-s002.zip › Dataset/airplane/13.png]

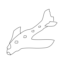

Supplement: Supplemental Information 2 [file peerj-cs-09-1186-s002.zip › Dataset/airplane/14.png]

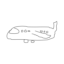

Supplement: Supplemental Information 2 [file peerj-cs-09-1186-s002.zip › Dataset/airplane/15.png]

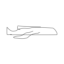

Supplement: Supplemental Information 2 [file peerj-cs-09-1186-s002.zip › Dataset/airplane/16.png]

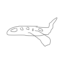

Supplement: Supplemental Information 2 [file peerj-cs-09-1186-s002.zip › Dataset/airplane/17.png]

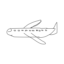

Supplement: Supplemental Information 2 [file peerj-cs-09-1186-s002.zip › Dataset/airplane/18.png]

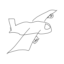

Supplement: Supplemental Information 2 [file peerj-cs-09-1186-s002.zip › Dataset/airplane/19.png]

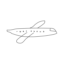

Supplement: Supplemental Information 2 [file peerj-cs-09-1186-s002.zip › Dataset/airplane/2.png]

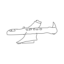

Supplement: Supplemental Information 2 [file peerj-cs-09-1186-s002.zip › Dataset/airplane/20.png]

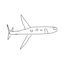

Supplement: Supplemental Information 2 [file peerj-cs-09-1186-s002.zip › Dataset/airplane/21.png]

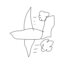

Supplement: Supplemental Information 2 [file peerj-cs-09-1186-s002.zip › Dataset/airplane/22.png]

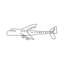

Supplement: Supplemental Information 2 [file peerj-cs-09-1186-s002.zip › Dataset/airplane/23.png]

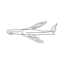

Supplement: Supplemental Information 2 [file peerj-cs-09-1186-s002.zip › Dataset/airplane/24.png]

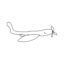

Supplement: Supplemental Information 2 [file peerj-cs-09-1186-s002.zip › Dataset/airplane/25.png]

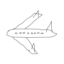

Supplement: Supplemental Information 2 [file peerj-cs-09-1186-s002.zip › Dataset/airplane/26.png]

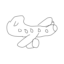

Supplement: Supplemental Information 2 [file peerj-cs-09-1186-s002.zip › Dataset/airplane/27.png]

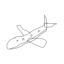

Supplement: Supplemental Information 2 [file peerj-cs-09-1186-s002.zip › Dataset/airplane/28.png]

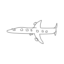

Supplement: Supplemental Information 2 [file peerj-cs-09-1186-s002.zip › Dataset/airplane/29.png]

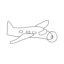

Supplement: Supplemental Information 2 [file peerj-cs-09-1186-s002.zip › Dataset/airplane/3.png]

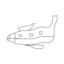

Supplement: Supplemental Information 2 [file peerj-cs-09-1186-s002.zip › Dataset/airplane/30.png]

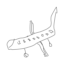

Supplement: Supplemental Information 2 [file peerj-cs-09-1186-s002.zip › Dataset/airplane/31.png]

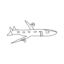

Supplement: Supplemental Information 2 [file peerj-cs-09-1186-s002.zip › Dataset/airplane/32.png]

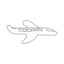

Supplement: Supplemental Information 2 [file peerj-cs-09-1186-s002.zip › Dataset/airplane/33.png]

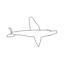

Supplement: Supplemental Information 2 [file peerj-cs-09-1186-s002.zip › Dataset/airplane/34.png]

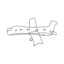

Supplement: Supplemental Information 2 [file peerj-cs-09-1186-s002.zip › Dataset/airplane/35.png]

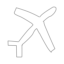

Supplement: Supplemental Information 2 [file peerj-cs-09-1186-s002.zip › Dataset/airplane/36.png]

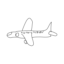

Supplement: Supplemental Information 2 [file peerj-cs-09-1186-s002.zip › Dataset/airplane/37.png]

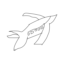

Supplement: Supplemental Information 2 [file peerj-cs-09-1186-s002.zip › Dataset/airplane/38.png]

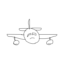

Supplement: Supplemental Information 2 [file peerj-cs-09-1186-s002.zip › Dataset/airplane/39.png]

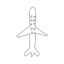

Supplement: Supplemental Information 2 [file peerj-cs-09-1186-s002.zip › Dataset/airplane/4.png]

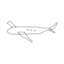

Supplement: Supplemental Information 2 [file peerj-cs-09-1186-s002.zip › Dataset/airplane/40.png]

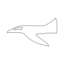

Supplement: Supplemental Information 2 [file peerj-cs-09-1186-s002.zip › Dataset/airplane/41.png]

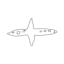

Supplement: Supplemental Information 2 [file peerj-cs-09-1186-s002.zip › Dataset/airplane/42.png]

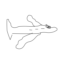

Supplement: Supplemental Information 2 [file peerj-cs-09-1186-s002.zip › Dataset/airplane/43.png]

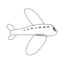

Supplement: Supplemental Information 2 [file peerj-cs-09-1186-s002.zip › Dataset/airplane/44.png]

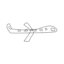

Supplement: Supplemental Information 2 [file peerj-cs-09-1186-s002.zip › Dataset/airplane/45.png]

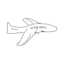

Supplement: Supplemental Information 2 [file peerj-cs-09-1186-s002.zip › Dataset/airplane/46.png]

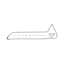

Supplement: Supplemental Information 2 [file peerj-cs-09-1186-s002.zip › Dataset/airplane/47.png]

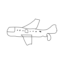

Supplement: Supplemental Information 2 [file peerj-cs-09-1186-s002.zip › Dataset/airplane/48.png]

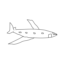

Supplement: Supplemental Information 2 [file peerj-cs-09-1186-s002.zip › Dataset/airplane/49.png]

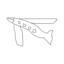

Supplement: Supplemental Information 2 [file peerj-cs-09-1186-s002.zip › Dataset/airplane/5.png]

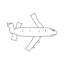

Supplement: Supplemental Information 2 [file peerj-cs-09-1186-s002.zip › Dataset/airplane/50.png]

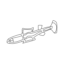

Supplement: Supplemental Information 2 [file peerj-cs-09-1186-s002.zip › Dataset/airplane/51.png]

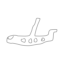

Supplement: Supplemental Information 2 [file peerj-cs-09-1186-s002.zip › Dataset/airplane/52.png]

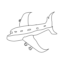

Supplement: Supplemental Information 2 [file peerj-cs-09-1186-s002.zip › Dataset/airplane/53.png]

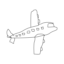

Supplement: Supplemental Information 2 [file peerj-cs-09-1186-s002.zip › Dataset/airplane/54.png]

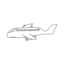

Supplement: Supplemental Information 2 [file peerj-cs-09-1186-s002.zip › Dataset/airplane/55.png]

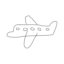

Supplement: Supplemental Information 2 [file peerj-cs-09-1186-s002.zip › Dataset/airplane/56.png]

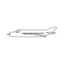

Supplement: Supplemental Information 2 [file peerj-cs-09-1186-s002.zip › Dataset/airplane/57.png]

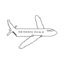

Supplement: Supplemental Information 2 [file peerj-cs-09-1186-s002.zip › Dataset/airplane/58.png]

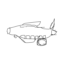

Supplement: Supplemental Information 2 [file peerj-cs-09-1186-s002.zip › Dataset/airplane/59.png]

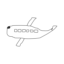

Supplement: Supplemental Information 2 [file peerj-cs-09-1186-s002.zip › Dataset/airplane/6.png]

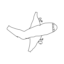

Supplement: Supplemental Information 2 [file peerj-cs-09-1186-s002.zip › Dataset/airplane/60.png]

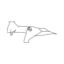

Supplement: Supplemental Information 2 [file peerj-cs-09-1186-s002.zip › Dataset/airplane/61.png]

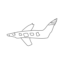

Supplement: Supplemental Information 2 [file peerj-cs-09-1186-s002.zip › Dataset/airplane/62.png]

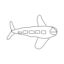

Supplement: Supplemental Information 2 [file peerj-cs-09-1186-s002.zip › Dataset/airplane/63.png]

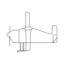

Supplement: Supplemental Information 2 [file peerj-cs-09-1186-s002.zip › Dataset/airplane/64.png]

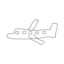

Supplement: Supplemental Information 2 [file peerj-cs-09-1186-s002.zip › Dataset/airplane/65.png]

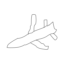

Supplement: Supplemental Information 2 [file peerj-cs-09-1186-s002.zip › Dataset/airplane/66.png]

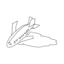

Supplement: Supplemental Information 2 [file peerj-cs-09-1186-s002.zip › Dataset/airplane/67.png]

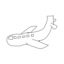

Supplement: Supplemental Information 2 [file peerj-cs-09-1186-s002.zip › Dataset/airplane/68.png]

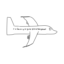

Supplement: Supplemental Information 2 [file peerj-cs-09-1186-s002.zip › Dataset/airplane/69.png]

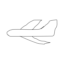

Supplement: Supplemental Information 2 [file peerj-cs-09-1186-s002.zip › Dataset/airplane/7.png]

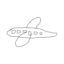

Supplement: Supplemental Information 2 [file peerj-cs-09-1186-s002.zip › Dataset/airplane/70.png]

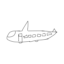

Supplement: Supplemental Information 2 [file peerj-cs-09-1186-s002.zip › Dataset/airplane/71.png]

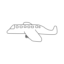

Supplement: Supplemental Information 2 [file peerj-cs-09-1186-s002.zip › Dataset/airplane/72.png]

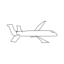

Supplement: Supplemental Information 2 [file peerj-cs-09-1186-s002.zip › Dataset/airplane/73.png]

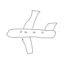

Supplement: Supplemental Information 2 [file peerj-cs-09-1186-s002.zip › Dataset/airplane/74.png]

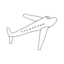

Supplement: Supplemental Information 2 [file peerj-cs-09-1186-s002.zip › Dataset/airplane/75.png]

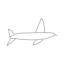

Supplement: Supplemental Information 2 [file peerj-cs-09-1186-s002.zip › Dataset/airplane/76.png]

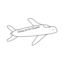

Supplement: Supplemental Information 2 [file peerj-cs-09-1186-s002.zip › Dataset/airplane/77.png]

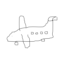

Supplement: Supplemental Information 2 [file peerj-cs-09-1186-s002.zip › Dataset/airplane/78.png]

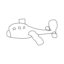

Supplement: Supplemental Information 2 [file peerj-cs-09-1186-s002.zip › Dataset/airplane/79.png]

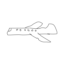

Supplement: Supplemental Information 2 [file peerj-cs-09-1186-s002.zip › Dataset/airplane/8.png]

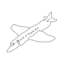

Supplement: Supplemental Information 2 [file peerj-cs-09-1186-s002.zip › Dataset/airplane/80.png]

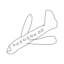

Supplement: Supplemental Information 2 [file peerj-cs-09-1186-s002.zip › Dataset/airplane/9.png]

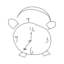

Supplement: Supplemental Information 2 [file peerj-cs-09-1186-s002.zip › Dataset/alarm clock/100.png]

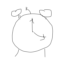

Supplement: Supplemental Information 2 [file peerj-cs-09-1186-s002.zip › Dataset/alarm clock/101.png]

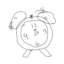

Supplement: Supplemental Information 2 [file peerj-cs-09-1186-s002.zip › Dataset/alarm clock/102.png]

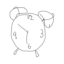

Supplement: Supplemental Information 2 [file peerj-cs-09-1186-s002.zip › Dataset/alarm clock/103.png]

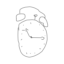

Supplement: Supplemental Information 2 [file peerj-cs-09-1186-s002.zip › Dataset/alarm clock/104.png]

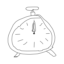

Supplement: Supplemental Information 2 [file peerj-cs-09-1186-s002.zip › Dataset/alarm clock/105.png]

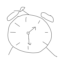

Supplement: Supplemental Information 2 [file peerj-cs-09-1186-s002.zip › Dataset/alarm clock/106.png]

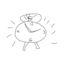

Supplement: Supplemental Information 2 [file peerj-cs-09-1186-s002.zip › Dataset/alarm clock/107.png]

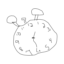

Supplement: Supplemental Information 2 [file peerj-cs-09-1186-s002.zip › Dataset/alarm clock/108.png]

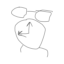

Supplement: Supplemental Information 2 [file peerj-cs-09-1186-s002.zip › Dataset/alarm clock/109.png]

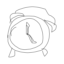

Supplement: Supplemental Information 2 [file peerj-cs-09-1186-s002.zip › Dataset/alarm clock/110.png]

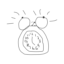

Supplement: Supplemental Information 2 [file peerj-cs-09-1186-s002.zip › Dataset/alarm clock/111.png]

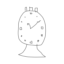

Supplement: Supplemental Information 2 [file peerj-cs-09-1186-s002.zip › Dataset/alarm clock/112.png]

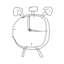

Supplement: Supplemental Information 2 [file peerj-cs-09-1186-s002.zip › Dataset/alarm clock/113.png]

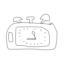

Supplement: Supplemental Information 2 [file peerj-cs-09-1186-s002.zip › Dataset/alarm clock/114.png]

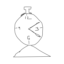

Supplement: Supplemental Information 2 [file peerj-cs-09-1186-s002.zip › Dataset/alarm clock/115.png]

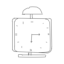

Supplement: Supplemental Information 2 [file peerj-cs-09-1186-s002.zip › Dataset/alarm clock/116.png]

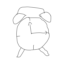

Supplement: Supplemental Information 2 [file peerj-cs-09-1186-s002.zip › Dataset/alarm clock/117.png]

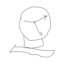

Supplement: Supplemental Information 2 [file peerj-cs-09-1186-s002.zip › Dataset/alarm clock/118.png]

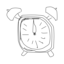

Supplement: Supplemental Information 2 [file peerj-cs-09-1186-s002.zip › Dataset/alarm clock/119.png]
